# Amadou as a Local Haemostatic in Military Surgery

**Published:** 1916-03

**Authors:** 


**Amadou as a Local Haemostatic in Military Surgery.** Henry Reynes Le Progres Med., Nov. 1915. Amadou is translated as German tinder, spunk, touch wood and amadow. It is the polyporus formentarius, a fungus of oaks and beeches, long used as a vulnerary and, impregnated with nitre, for the old moxas. Slices 8-12 c.m. long and about 2 transversely, are cut from the middle portion of the fungus, soaked and beaten until they become supple. Its haemostatic action may be reinforced by absorption of ferric chlorid or, to prevent irritation, by treatment with ferric chlorid afterward neutralized by soda solution. The author has employed it, especially for bone haemorrhage, in its ordinary form, sterilized with instruments at 130-140 C. dry heat. It soaks up blood, as well as preventing haemorrhage, does not adhere and may be removed after 1-2 days.

